# Wellbeing Impacts of City Policies for Reducing Greenhouse Gas Emissions

**DOI:** 10.3390/ijerph111212312

**Published:** 2014-11-28

**Authors:** Rosemary Hiscock, Pierpaolo Mudu, Matthias Braubach, Marco Martuzzi, Laura Perez, Clive Sabel

**Affiliations:** 1 School of Geographical Sciences, University of Bristol, University Road, Clifton, Bristol BS8 1SS, UK; E-Mail: c.sabel@bristol.ac.uk; 2 WHO Regional Office for Europe, European Centre for Environment and Health, Bonn office, Platz der Vereinten Nationen 1, 53113 Bonn, Germany; E-Mails: mudup@ecehbonn.euro.who.int (P.M.); mbr@ecehbonn.euro.who.int (M.B.); martuzzim@ecehbonn.euro.who.int (M.M.); 3 Swiss Tropical and Public Health Institute, Socinstr. 57, Basel 4051, Switzerland; E-Mail: l.perez@unibas.ch

**Keywords:** climate change, greenhouse gas emissions, cities, wellbeing

## Abstract

To mitigate climate change, city authorities are developing policies in areas such as transportation, housing and energy use, to reduce greenhouse gas emissions. In addition to their effects on greenhouse gas emissions, these policies are likely to have consequences for the wellbeing of their populations for example through changes in opportunities to take physical exercise. In order to explore the potential consequences for wellbeing, we first explore what ‘wellbeing’ is and how it can be operationalized for urban planners. In this paper, we illustrate how wellbeing can be divided into objective and subjective aspects which can be measured quantitatively; our review of measures informs the development of a theoretical model linking wellbeing to policies which cities use to reduce greenhouse gas emissions. Finally, we discuss the extent to which the links proposed in the conceptual model are supported by the literature and how cities can assess wellbeing implications of policies.

## 1. Introduction

This paper is part of URGENCHE (Urban Reduction of Greenhouse Gas Emissions in China and Europe), a European Commission funded project to assess the health and wellbeing implications of city policies for reducing greenhouse gas (GHG) emissions. The assessment is based on scenarios within seven case study cities and aims to identify the effects that municipal housing, transport and energy measures to reduce GHG emissions would have on health and wellbeing by the year 2020. While health related results are beginning to be appear elsewhere [1], this paper focuses on wellbeing firstly because, despite being less well understood than health, wellbeing is a desirable goal in itself [2] and secondly it is critical for future wellbeing that we preserve an environment meets basic needs such as water and clean air [3]. Climate change threatens this and even where basic needs can be met it is likely to increase psychological stressors through, for example, unpredictable weather patterns and migration [4]. To implement mitigation policies the agreement of the people is needed. Consensus is more likely if current wellbeing is not compromised and if there is a shared understanding of the possible co-benefits compared with dis-benefits of policies [4,5].

WHO Regional Office for Europe’s proposed definition is that wellbeing “comprises an individual’s experience of their life as well as a comparison of life circumstances with social norms and values” [6]. In addition to academics, “wellbeing” is of interest to charities [7], non-governmental organizations [8,9] and governments [10] in order to understand how society is “doing” [11]. Given the aim of URGENCHE is to provide quantitative estimates of consequences of implementing GHG reduction policies, wellbeing is primarily operationalized here through scales and indices rather than through qualitative work. 

The project’s empirical limitation to the real actions implemented in cities has meant that URGENCHE assessments of wellbeing are restricted to the GHG interventions under consideration by the project cities: it therefore does not include urban planning polices such as creating green space, or increasing housing density as they were not chosen by the URGENCHE cities as part of the project. However, this does not indicate that such policies would not have wellbeing effects. 

The objectives of this paper were firstly to develop a conceptual framework of wellbeing relevant to greenhouse gas reduction policies and secondly to operationalize the introduced concepts in order to guide the study of the effects of GHG policies on wellbeing. The conceptual model emerged from both *a priori* and *a posteriori* processes: from the authors’ previous experience of the literature in this area and their experiences of working with cities for URGENCHE and also from evidence collected through our compilation of literature (details provided later) undertaken specifically for the project; thus the authors concur with Williamson that the distinction between the two processes is superficial [12].

## 2. A Conceptualization of Wellbeing Relevant to Greenhouse Gas Reduction Policies

In this section we make a very limited introduction to the concept of wellbeing in order to introduce the reader to the concept. If the reader is interested in a more critical study of the concept we suggest that they peruse some of the following references [13,14,15,16,17,18]. 

The concept of wellbeing is currently under discussion and development but it is generally recognized that it involves subjective and objective components [6,19,20,21,22]. The subjective aspects of wellbeing involve the first part of the WHO Regional Office for Europe definition-an individual’s experiences [6] including “psychological functioning and affective states” [19]. Objective wellbeing involves “a comparison of life circumstances with social norms and values” [6]. Thus wellbeing can be seen as enacted on both an individual subjective level and a social objective level. In addition to the subjective/objective dimension, wellbeing is also theorized in terms of hedonic/eudemonic dimensions: “hedonic” wellbeing involves happiness, pleasure and enjoyment where wellbeing is achieved by avoiding pain and seeking pleasure, and “eudemonic” wellbeing which is achieved through finding purpose, meaning and fulfillment [20,21,23].

WHO’s 1948 Constitution defines health as “a state of complete physical, mental and social well-being and not merely the absence of disease or infirmity” [24] and thus views disease and infirmity as one end of a spectrum and wellbeing as the opposite end; thus health is both absence of disease and presence of wellbeing. It appears that health is both a determinant and an outcome of wellbeing or they are mutually constitutive factors [19]. The definition also reflects the Cartesian philosophical idea that the mind and the body represent different functioning systems [25] and thus physiology (or physical health) can be separated from subjective wellbeing. Despite the shortcomings of this approach [25], for clarity we will use “health” in this restrictive sense, referring to absence of disease and directly measurable outcomes such as life expectancy. Although the WHO definition of health (which encompasses wellbeing) is from 1948, it is only in the last three decades that discussion on the need to measure different aspects of non-physical health and wellbeing has gained prominence. 

The term “wellbeing” is used in association with “positive mental health” [26,27]. In our conceptual model we have therefore considered studies that measure mental health to be measuring wellbeing; however it must be noted that wellbeing is more than just absence of psychological distress [22,28,29]. The WHO Regional Office for Europe argues that adverse outcomes resulting from lack of wellbeing are mainly depression and other mental illnesses and thus subjective wellbeing should be measured because negative outcomes lead to costs for health services [19]. Mental disorders involve the inability to manage thoughts, emotions, behaviors and interactions with others and can be caused by social, cultural, economic, political and environmental factors; these include national policies, social protection, living standards, working conditions, and community social supports [30]. Mental health, and in particular depression, has been measured in a variety of contexts, both on a continuous scale, other times as a dichotomy (such as depressed *vs.* not depressed). Self-assessed health tends to reflect respondents’ mental health and thus is also an indicator of mental health [31]. Thus, mental health can be measured in a variety of different ways. Some studies have however used “wellbeing” itself as an outcome rather than mental health but again there are many available measures.

There is no single ideal wellbeing measurement [19] and through a critical examination of the operationalization of wellbeing by various measures, we intend to illustrate further the concept of wellbeing through issues that arise. The review of wellbeing measures below shows that many current measures reflect different understandings of wellbeing and often confuse objective and subjective wellbeing; ideally self-complete scales assess subjective wellbeing, while wellbeing indices include objectively measured environmental and personal conditions which are likely to lead to high subjective wellbeing (There is considerable discussion on how indices and scales should be differentiated. For the purposes of this article, a scale is multiple items usually measuring one factor, using a common set of responses (e.g., agree strongly, agree slightly, disagree slightly, disagree strongly), whereas an index is where multiple indicators are amassed). Although some have argued that a fuzzy definition of wellbeing is helpful in that it allows people to use wellbeing for their own purposes [32], we suggest that conflating objective and subjective wellbeing lays the field open to criticism as the direction of causality is difficult to infer. 

In this paper, we seek to help academics and policy makers choose how to measure subjective and objective wellbeing as potential outcomes of their policies and thus we review subjective wellbeing measures and refer to some of the available general objective wellbeing measures in advance of concentrating on measure of objective wellbeing that are more likely to be affected by GHG reduction policies. Our review also informed the development of our conceptual model.

### 2.1. Subjective Wellbeing Measures

There are many subjective wellbeing scales (see [33,34,35] for more detailed overviews) and we have space here only to discuss five examples which are intended to illustrate the breadth of possible measures and help the reader to reflect upon what they believe the concept of wellbeing to be. We encourage the reader to think about more than just statistical validity and to instead consider carefully the items used in the scale. Firstly we discuss the WHO-5 scale which, after careful consideration of the items found in a number of scales, was used in the development of our conceptual model of environmental impacts on wellbeing. Other approaches to wellbeing, such as satistaction, of course have merit and have been used in peer reviewed and well-received studies of wellbeing so we continue by describing selected alternative measurement tools and their approach to define wellbeing, illustrating the diversity of measures and concepts developed and their strengths and weaknesses.

The WHO-5 Wellbeing scale [36] was developed specifically to measure wellbeing. It has been translated into many languages and has been successfully statistically validated in a variety of populations [37,38,39,40,41]. It is practical to use, consisting of only five questions. Respondents are asked to rate their wellbeing on a six point scale over the last two weeks, thus it is not just measuring momentary feelings. The five items capture hedonic aspects of wellbeing (cheerfulness and good spirits, the abilities to relax, feel rested and be active) and the eudemonic aspect in “experiencing life as full of interest”.

A possible disadvantage of WHO-5 is that it was originally developed to measure wellbeing in diabetes patients and thus the wellbeing measured could be “wellbeing despite disease” which could be problematic in a healthy population. The recognition that people with physical health issues can experience high levels of wellbeing is of significance in itself and lends credence to the notion that wellbeing is more than just one end of a spectrum with disease at the other end. Measures of physical health are often included in objective wellbeing indices, as is socioeconomic status; those measuring objective wellbeing without also measuring subjective wellbeing are making assumptions that those in poor physical health and socioeconomically disadvantaged groups are not experiencing high levels of wellbeing, but both these groups can report good levels of subjective wellbeing [40,42].

Nevertheless the WHO-5 scale has however now been successfully used in a variety of other settings in addition to diabetes research [43,44,45]. Notably, in the light of GHG reduction policies, the WHO-5 is included in the European Quality of Life Survey 2012 [45], which enables the measurement of linkages between external conditions (such as social, occupational, and environmental domains) and subjective wellbeing [27]. 

Another well-known successfully statistically validated scale measuring subjective wellbeing is the WEMWBS Warwick-Edinburgh Mental Well-Being Scale [26,46,47]. The fourteen WEMWBS items allow the scale to widen the definition of wellbeing but are more onerous for respondents to complete. There is now a shorter seven item version available (SWEMWBS or short WEMWBS) [48]. The items are “I’ve been feeling optimistic about the future”, “I’ve been feeling useful”, “I’ve been feeling relaxed”, “I’ve been dealing with problems well”, “I’ve been thinking clearly”, “I’ve been feeling close to other people” and “I’ve been able to make up my own mind about things”. Thus the SWEMWBS is more focused on cognitive, rather than affective, aspects than WHO-5 and deliberately includes relationships with other people as part of wellbeing itself. There is consequently the possibility that this formulation of wellbeing may be less likely to apply universally to all people because perceptions of social ties with other people is, at least to some extent, culturally patterned. Both the WHO-5 and SWEMWBS phrase all items positively, in contrast to scales measuring mental illness, but the SWEMWBS scale refers to concepts that are not positive such as “dealing with problems” and “having to make decisions” which may not carry positive valence. 

A different approach focuses on satisfaction with life, which can be operationalized as a single item or as a tool covering various elements. Observation of the extensive use of life satisfaction in international surveys [34] and concerns about the theoretical and empirical underpinnings of the links between hedonism and eudemonism and health [49] (which have some potential to be addressed in this paper or by theoretical and empirical research inspired by this paper), led the WHO Regional Office for Europe to adopt a single item on satisfaction with life as the core indicator for monitoring subjective wellbeing in the newly established health policy (Health 2020) [50]. An example of a more complex tool using multiple items is the successfully statistically validated “satisfaction with life scale” developed in the US [51,52]. Diener’s definition of wellbeing defines subjective wellbeing as “how people evaluate their lives” [53,54]. Thus the five items (“In most ways my life is close to my ideal”, “the conditions of my life are excellent”, “I am satisfied with my life”, “So far I have gotten the important things I want in life” and “If I could live my life over, I would change almost nothing”) are all about evaluation rather than general feelings. The comparative element in this scale may be too close to objective wellbeing as it is likely to shift a person’s thoughts towards their life conditions rather than feelings: it would be hard for a person with low socioeconomic status or poor social relationships to acquire a high score (see [42]). Furthermore as a term, “satisfaction” is highly subjective and charged with different meanings according to context; for example the rating “satisfactory” has recently changed from being acceptable to “requires improvement” for English school inspections [55]. 

The developers of a Dutch subjective wellbeing scale, the SPF-IL scale [56] noted that people tend to assess wellbeing affectively (as in the WHO-5) and cognitively (as in SWEMWBS) and attempts to focus respondents answers to reflect both by asking respondents about their experiences rather than general statements. These authors used the theory of Social Production Functions to develop items intended to measure how well respondents reach goals of affection, behavioral confirmation, status, comfort and stimulation. However, the scale encompasses fifteen items, and in the validation study there were many missing values for the status items, suggesting this scale did not appeal to respondents. 

Finally, the UK Office of National Statistics (ONS), uses four experimental questions on subjective wellbeing: general life satisfaction; feeling that actions are worthwhile; happiness yesterday; and anxiety yesterday [57]. These questions cover both hedonic and eudemonic aspects of wellbeing. The four questions are analyzed separately rather than being combined into a scale. Eleven point scales are used for responses. However, generally the upper limit for Likert scale responses is seven [58] due to limitations in human’s ability to visualize larger numbers [59]. Another possible criticism is that the questions only consider as far back as yesterday whereas the WHO-5 scale answers over a longer time period (two weeks). Subjective wellbeing when measured over time is surprisingly stable [60]. To be certain to be measuring this more stable concept the WHO-5 scale acknowledges the temporal context of wellbeing by asking about experiences over the duration of a two week period. Thus the ONS questions may reflect particular life events rather than a more stable feeling.

These five examples of subjective wellbeing scales provide some understanding and evaluation of the breadth of approaches to the quantitative measurement of subjective wellbeing. Whether wellbeing is primarily emotional or cognitive, whether relationships with other people are a predictor of or part of wellbeing and the universality of the wellbeing measured is likely to vary with the scale used. Only the WHO-5 and ONS clearly include both hedonic and eudemonic aspects. The cognitive assessments of SWEMWBS and evaluations of the “satisfaction with life” scale may occur from a person either reflecting that they have positive life circumstances which lead to (or result from) subjective wellbeing rather than subjective wellbeing itself (as measured by the WHO-5 scale). However, irrespective of the conceptual differences of the tools, there is little likelihood that cities regularly collect data on any of these; nevertheless the WHO-5 has the additional advantage of having been included within the EQLS 2012 [45], enabling associations between wellbeing and a variety of other dimensions to be tested, thus making it a pragmatic choice for theory development which can then be empirically tested.

### 2.2. Objective Wellbeing Indices

There has been growing interest in developing “objective wellbeing” [20] or “livability” [61] measures often at a national scale [6,30]. A range of projects, such as WHO Regional Office for Europe’s consultation on targets and indicators for wellbeing [49,62] and indices attempt to measure objective wellbeing, such as the OECD Better Life Index [63] and the Oxfam’s Humankind Index [7]. National measures are also being developed, for example, by Gallup (US) [64], Istat (Italy) [65], Health Utilities Inc. (Canada) [66], INSEE (France) [67] and ONS (UK) [68]. The Dutch “Leefbaarometer”, a survey run every five years, offers detailed information on a list of wellbeing issues at a zip code scale [69,70]. Commonly the indices include health, health related behaviors, sustainability and environment, socioeconomic status and social support. Social factors are more to the fore in comparison to the individualistic style of items included in subjective wellbeing scales. More detail is now provided on sustainability and environment, socioeconomic status and social support with the exception of health and health behaviors because health has been discussed previously and physical health, although relevant, is outside the scope of this paper.

#### 2.2.1. Sustainability and Environment

Often progress is measured in terms of GDP [8], either because wealth is seen as an end as itself or because economic wellbeing is recognized as important [32]. Wellbeing is not just about economics and striving to increase wellbeing in general, rather than GDP, has been argued to lead to a more sustainable future [8]. However, there is potential tension between wellbeing and sustainability [71]: either there is a compromise on wellbeing now to improve wellbeing for future generations or there is no compromise now but there but there will be severe reduction in wellbeing for future generations [72]. Wellbeing thus needs to be considered temporally (regarding future catastrophe) and spatially (regarding environments we inhabit).

Temporal conceptualizations of wellbeing take into account changes in wellbeing over time and the difference between short and long term goals [73]. It is an issue for city planners that citizens may think of their own short term wellbeing rather than the wellbeing of future generations when they evaluated GHG reduction options [74]. It may also be hard for people to change their life patterns which provide short term comfort, such as car use, for long term environmental sustainability [75]. 

Because many of the factors affecting wellbeing are spatially structured (as for example they involve contextual variables pertaining to local communities, such as cohesion), the environment and location-specific factors have a much larger influence in determining wellbeing than previously thought for example through natural environment characteristics, services available, and congeniality and socioeconomic status of the population [76]. It has been argued that these are as important as individual socio-economic or demographic factors [77]. The significance that respondents attribute to wellbeing and wellbeing scores can vary depending on the cultural and political context [6,78,79] but nevertheless there are sufficient synergies for comparisons between areas to be valid [42]. A spatial conceptualization can be described by maps or a lived experience of a “place” [80]) and need to bear in mind geographic scale that ranges from the individual to more aggregate levels [81,82]. Objective social indicators collected for well-defined administrative units or areas are unlikely to represent the territorial base of an individual’s wellbeing [83]. Neighborhood satisfaction, for example, will depend on the effective space “inhabited” by an individual, and be meaningful in relation to that space, rather than administrative units [84]. 

Thus, consideration of climate and environmental conditions is critical when analyzing objective wellbeing [77,85] and therefore, many objective wellbeing indices include parameters related to the provision of the population with adequate environmental services and conditions (e.g., the Dutch Leefbarometer covers housing, noise and green spaces, the OECD Better Life Index includes an environmental component covering air pollution and water quality and a housing component covering rooms per person and dwelling facilities, and the Humankind Index from Oxfam covers green spaces, clean and healthy environments, and having an affordable and decent home). 

The literature of sustainability and wellbeing has been drawn upon in the conceptual model in the second part of this paper—although, for simplicity, the model underrepresents concepts of time and space.

#### 2.2.2. Socioeconomic Status

One common component of objective wellbeing that has relevance for cities but warrants particular discussion is socioeconomic status (SES). There are concerns that a focus on the fuzzy concept of wellbeing may reduce the priority to decrease inequalities [86]. Nevertheless policies to reduce disadvantage should also improve wellbeing because low SES is associated with lower subjective wellbeing whereas affluence, however, is not strongly associated with higher subjective wellbeing especially when subjective wellbeing is measured in terms of stable affect rather than in terms of satisfaction [42]. This may be because materialism is associated with poorer wellbeing [72]. Thus in the urban context, it is important to be able to differentiate the wellbeing effects of policies on citizens of different socioeconomic levels. 

Given that residential choices are often affected by socioeconomic status, there are spatial injustices in access to services, geographical variations in standards of living and exposure to pollution or noise, and discrepancies in access to therapeutic landscapes and health-promoting urban features [87]. Furthermore there needs to be a balance between direct effects of a policy on wellbeing and indirect effects for example through economic growth [88], as a factory may reduce health and wellbeing of the local community through air pollution but may increase health and wellbeing through employment.

#### 2.2.3. Social Relationships

Social relationships are often viewed by social scientists through the concept of social capital. Social capital involves, in addition to positive informal social relationships, participation in clubs and voluntary associations, voting patterns and social trust [89]. Social capital can be conceived of as a kind of aggregate level of eudemonic wellbeing (and perhaps overlapping with the Chinese concept of “harmonious society” [90]). 

### 2.3. Combined Measures

Some instruments, for example WHO QOL-BREF [91], EUROHIS-QOL [92] and the Happy Planet Index [8] combine subjective and objective wellbeing measures and subjective wellbeing is often included as one measure within what are ostensibly objective wellbeing indices (e.g., [65]). This is a problematic procedure because the two are very different concepts and should be kept separate in order to study the intricacies of the relationships between them. Similarly the Personal Wellbeing Index [93,94], although often described as a subjective wellbeing index, has a domain based approach to wellbeing (it asks about levels of satisfaction with a list of specific items (standard of living, health, achievement, relationships, safety, community and future security)). Statistical validation of the PWI shows only moderate correlation between domains [94]. Thus a domain approach may be unsatisfactory as an attempt to measure global wellbeing. A global measure is important because generating a comprehensive list of contributing domains is difficult and also domains on the list, are likely to change over time and even if they remain relevant their importance may change [21]. 

**Figure 1:**
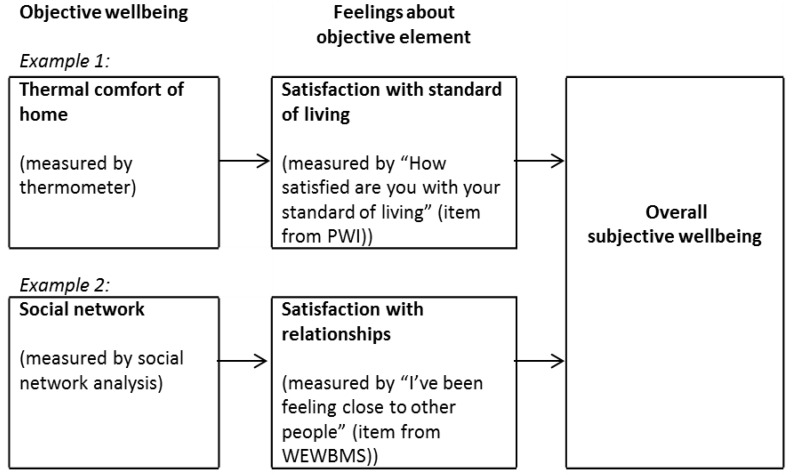
The continuum between objective and subjective wellbeing: an example with thermal comfort and social networks.

Furthermore given the PWI is asking about satisfaction with various domains, it is arguably measuring a concept which is on the pathway moving from objective wellbeing to subjective wellbeing rather than subjective wellbeing itself (Figure 1). The three steps on the pathway (illustrated in Figure 1) are cognizant with the three policy relevant accounts of wellbeing: objective lists, preferences satisfaction and mental states [21,95]. Two domains are provided in Figure 1 as examples of differentiation between ‘pure’ objective wellbeing, “pure” subjective wellbeing and measures between these two poles. The example domains are thermal comfort of the home and social networks together with examples of measurements. Objectively measurable externalities are evaluated internally and then an overall feeling of subjective wellbeing is likely to arise from the merging of various domains. This pathway is relevant to our topic because for a GHG reduction policy to have a positive effect on wellbeing it will need to have a positive effect on people’s feelings as well as on objective measures. For example a policy that reduces car use may increase active mobility and reduce pollution, but it is not necessarily the case that those affected will feel positive about the forced change. 

In order to operationalize the concept of wellbeing for the purpose of URGENCHE research on urban policies for GHG emission reduction, we summarize our review of subjective and objective wellbeing measures: subjective wellbeing can be measured in a short scale which includes hedonic and eudemonic items but not evaluative and cognitive items whereas objective wellbeing should be measured in terms of tangible independently observed characteristics such as medical conditions, socioeconomic status and characteristics of the environment. From our review of subjective wellbeing indices we conclude that the WHO-5 is a good basis for understanding subjective wellbeing because firstly it does not include feelings about objective elements, secondly it is a global measure rather than domain based, thirdly it is feelings based rather than cognitively based and fourthly it has already been used in a European wide survey [45] of environmental and social dimensions and wellbeing as demanded by the URGENCHE project. 

Our review of objective wellbeing indices suggests that socioeconomic status, sustainability, relationships and physical health are important aspects but objective wellbeing will need to be defined further for our purposes in the conceptual model. In order to develop this analysis of quantitative measures of wellbeing in the context of urban GHG policy, it is necessary to develop a conceptual model which integrates these approaches.

## 3. Development of the Conceptual Model within Policies for Reducing Greenhouse Gas Emissions

**Figure 2:**
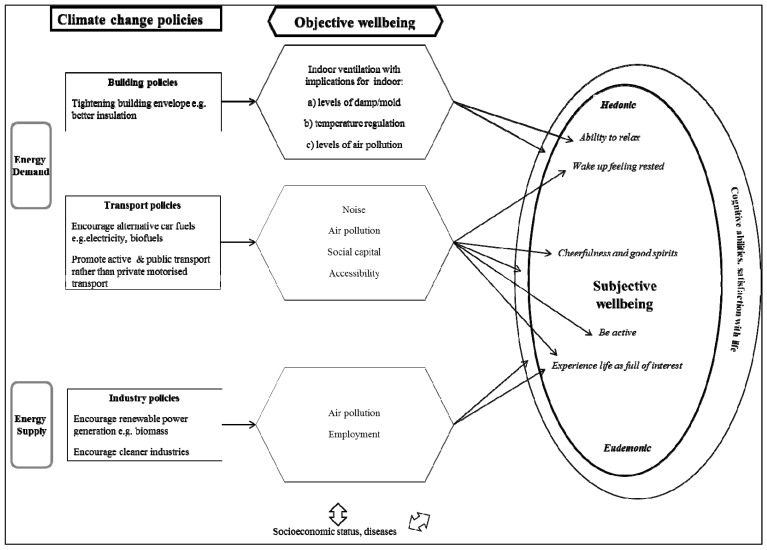
Conceptual model of some example policies to reduce greenhouse gas emissions and wellbeing.

A conceptual model connecting subjective wellbeing, through objective wellbeing, to the potential results of GHG reduction policies is provided in Figure 2. Our conceptual model was theory driven using the wellbeing framework of subjective *vs.* objective wellbeing and hedonic *vs.* eudemonic wellbeing and additionally informed by the overview of the literature on concepts relevant to greenhouse gas emission reduction policies and wellbeing described later. The arrows indicate suspected relationships, likely effects and potential consequences which are often implicit assumptions made by policy makers and academics but have not been previously articulated. The boxes on the left represent examples of urban policies applied to mitigate climate change; the hexagons in the middle represent effects of the policies on objective wellbeing, and in the oval on the right there are facets of subjective wellbeing as measured by the WHO-5 index. The purpose of the conceptual model is to guide cities as to the areas they need to think about when considering how a particular policy could affect wellbeing, but also suggests an analytical research framework for quantifying the potential wellbeing impacts of such policies. There is evidence, or at least discussion on each pathway in the academic literature, particularly the literature on sustainability and wellbeing [72], but more work is needed on the relative strengths of associations.

### 3.1. Climate Change Policies in the Conceptual Model

On the left of Figure 2 are some of the GHG reduction building, transport and energy generation policies that cities in URGENCHE wanted to include in modelling. All GHG reduction policies considered by cities are aimed either at energy supply, for example biomass production, or reducing energy demand, for example through tightening the building envelope. 

More context is now presented on the potential effects of and linkages between the GHG reduction policies and wellbeing. The following two sections describe the central part of the conceptual model (Figure 2) on how urban policies may affect environmental dimensions relevant for wellbeing. The effects are described in the order that they appear in the model; however many are strongly interconnected.

### 3.2. Objective Wellbeing Effects of GHG Reduction Policies in the Conceptual Model

In this section, objective wellbeing effects of policies on buildings, transport and industry are discussed.

#### 3.2.1. Building Policies and Objective Wellbeing

Housing policies on tightening building envelopes and improved insulation are likely to have positive implications for thermal comfort. However, studies have shown that a one-sided focus on energy saving without adequate consideration of ventilation rates may increase indoor pollution, dampness and mold growth, negatively affecting indoor environmental conditions [96,97]. Indoor air quality, dampness and mold growth are measurable conditions that, in addition to health, can also affect comfort and feelings of wellbeing [98]. 

Another potential pathway linking housing and energy efficiency with wellbeing could be the budget savings households would make by reduced heating bills, releasing this money for other household needs and thus affecting wellbeing indirectly. 

#### 3.2.2. Transport Policies and Objective Wellbeing

Transport policies are thought to affect wellbeing through ease of access to daily life destinations such as work, education, recreation and consumption, through the benefits of mobility itself (related to social relationships/social capital and physical activity), and externalities such as air and noise pollution [99]. 

Adequate access to a variety of destinations has been found to be important for objective wellbeing in terms of social capital, work opportunities and physical activity [99]. However, discouraging the use of private transport may reduce the accessibility of some destinations such as employment [100], cultural activities [101], green space and other destinations that engender physical activity and places to socialize [102]. Improvements in public transport and designing walkable neighborhoods [103,104] may mitigate this to some extent. However, it should be noted that the effects of accessibility may be superseded by socioeconomic status as disadvantaged areas in some cities may have many destinations on their doorstep whereas wealthy households who can afford one car per adult may commute in from great distances [105]. 

Social capital can be developed through encouraging active or public transport, for example through street connectivity, so that people spend more time in their local areas and through interacting with people on public transport and conversely people are more likely to walk if they have higher levels of social capital [106,107,108].

Finally, high numbers of petrol and diesel powered vehicles in urban settings may cause annoyance from air pollution and noise [72,109,110,111]. Promoting electric cars and active transport rather than petrol/diesel transport may mitigate the local environmental pollution associated with petrol and diesel use in urban traffic and reduce noise at the low speeds generally found in cities (although quieter cars travelling at speed may result in more accidents) [112].

#### 3.2.3. Industry Policies and Objective Wellbeing

More sustainable and effective energy generation and consumption patterns within the urban industrial sector could significantly reduce not only GHG emissions, but also the emission of air pollutants within a city. Similar to the traffic-related local pollution, this could be expected to have an impact on both health and wellbeing. 

Changes in energy supply and improved production technologies might not only affect energy efficiency alone, but also increase general productivity. This may affect productive and industrial activities and result in a net growth or decline in employment [113]. For example, employment decline could occur in cities where local heavy industry and energy production is outsourced to reduce CO_2_ emissions within the city itself; while new employment options could be generated through green economy investments in e.g., renewable energy technology or sustainable production [114]. It is possible that low-carbon economies may enable more jobs to be created than lost [115].

In summary, housing, transport and industry policies on reducing energy consumption and GHG emissions may have direct and indirect impacts on objective wellbeing though modification of housing conditions, air quality, social capital, accessibility and unemployment. The examples of employment and potential indoor problems show that the effects are not exclusively beneficial, indicating that such policies may have caveats and negative outcomes also. However, irrespective of the evaluation of the effects as positive or negative, these environmental dimensions of objective wellbeing will have further implications for individual subjective wellbeing as discussed below. 

### 3.3. Subjective Wellbeing Effects of GHG Reduction Policies in the Conceptual Model

The oval representing subjective wellbeing in the model provides an assessment of a “pure” form of subjective wellbeing, as the WHO-5 wellbeing score does not refer to intermediate domains such as health, personal relationships, environment or thought processes or the subjective and situated notion of satisfaction. The five WHO-5 items are included as part of wellbeing and graded by their reflection of hedonism and eudemonism. We suggest that building policies are perhaps more likely linked to hedonism and transport and industry to eudemonism in the following discussion. 

#### 3.3.1. Building Policies Implications Subjective Wellbeing (via Housing Conditions)

There is extensive literature on the importance of home and its meaning on people’s lives [116]. The ideal home may be a place of comfort to enable inhabitants to rest and relax. If housing conditions are poor—be it due to inadequate thermal comfort, dampness and indoor pollution or other factors-then a dwelling is less comfortable and it may be harder to relax [117]. This also applies to noise which may be generated by transport but largely affects people at home and strongly affects residential satisfaction, which is considered a component of overall life satisfaction and wellbeing [118], thus noise may a reduce the ability to relax and rest and to wake up feeling rested. Various studies have attempted to measure the extent to which noise causes “noise annoyance” or sleep disturbance as potential intermediary factors between noise and wellbeing [118,119,120,121,122,123,124]. 

#### 3.3.2. Transport Policy Implications for Subjective Wellbeing (via Active Transport, Social Capital and Air Pollution)

The objective wellbeing measure of accessibility and the greenhouse gas reduction policy of encouraging active transport are linked to the subjective wellbeing aspect of being active and realization of personal interests [125]. However, they potentially work in opposing directions, as encouraging active transport engenders increased physical activity (which is positive for good spirits and being active) [101] but reducing the use of other modes of transport could reduce the available venues for such activity to take place. 

The same is likely to apply to increased interpersonal contacts and social capital as a potential consequence of more active and public transport, which is likely to interact with cheerfulness and good spirits as well as being active and experiencing life full of interest [126,127,128]. However, unsafe neighborhoods where people are afraid to be out alone or after dark may counteract such a positive wellbeing effect and actually restrict the ability to relax, to be in good spirits, and to be active within the local neighborhood area [129]. 

However, no trade-offs would be expected for noise and air pollution, as these should be reduced by the GHG transport interventions and thus improve subjective wellbeing without negative side effects. Perceived levels of noise and air pollution are associated with life satisfaction and happiness [130,131]. More directly air pollution from SO_2_, NO_2_, PM_2.5_ and PM_10_, has been found to be associated with mental health [132] in addition to established detrimental physical health effects [110].

#### 3.3.3. Industry Policy Implications for Subjective Wellbeing (via Air Pollution and Employment)

In addition to reducing air pollution from transport, reducing air pollution from industry is likely to have beneficial effects on wellbeing [133] and additionally creation of a cleaner environment may be evidence of responsive governance reducing feelings of powerlessness and stigma among nearby residents [134,135]. 

The other objective wellbeing effect of industrial change through complying with GHG reduction policies identified is employment opportunities. Becoming unemployed is associated with poor mental health which tends to improve after regaining employment [136]. This is likely to reflect the “eudemonic” aspect of wellbeing [137] (activity level, experiencing life with full interest). Additionally subjective wellbeing effects of unemployment are likely to be related to changes in income [138]. 

In summary, there seem to be strong conceptual links between urban policies to reduce GHG emissions and wellbeing. It is, however, difficult to quantify this conceptual model, firstly because cities and other health authorities do not often collect WHO-5 or other subjective wellbeing measurements routinely and secondly because a quantitative assessment is often not feasible due to missing information on the nature and the extent of the relationships between urban dimensions and wellbeing. In the next section possible alternatives for wellbeing assessment are described, and other issues to note in the conceptualisation of wellbeing are addressed. GHG reduction interventions at an urban level could have some effect on wellbeing, but as indicated above these could be compensated or counteracted by other factors, such as economic contraction, thus hiding the potential effects of the interventions. Bearing in mind these difficulties, we present below a potential methodological approach for conducting a wellbeing assessment that we have developed for URGENCHE.

## 4. Quantification of the Theoretical Links between City Conditions and Wellbeing

Our conceptual model makes suggestions about links between wellbeing and GHG reduction emission policies. Quantification of these theoretical links would be useful for cities wishing to carry out a “wellbeing impact assessment” of policies in a similar way to a Health Impact Assessment (HIA). Literature on linkages between urban conditions (objective wellbeing) which might be affected by the policies in Figure 2, and associated subjective wellbeing which offers an indication to contextualize the conceptual framework and suggestions for further research and discussion, was compiled. As not much evidence was available using the concept of wellbeing itself, allied concepts of mental health and satisfaction were considered in addition to wellbeing, as potential outcomes considered. 

Each literature compilation was based on searches of the Web of Knowledge, PubMed and Google Scholar databases. For some of the linkages, very little research was found and Google itself was searched: we supplemented these findings with references known to the authors and for topics with little research, scanning of bibliographies. If an interesting article was found we conducted additional searches for similar articles. If a search did not appear to be generating relevant articles we truncated our evaluation of the papers found. The main searches were started in the traditional way by searching for key words, downloading articles and then searching for relevant papers looking first at the titles then the abstracts and then the full papers. Supplementary searches were made later where only relevant papers were downloaded to databases. The search terms used and references generated from the searches are presented in Table 1. Papers were included in our compilation if they presented statistics on quantitative associations between policy implications (or objective wellbeing) and subjective wellbeing.

**Table 1:** Searches terms and results.

Policy Area and Search Number	Search Terms *	Total Papers	Papers Providing Quantitative Assessment of Links
***BUILDINGS***			
1	“((damp/mold/mould) / (thermal comfort/(cold & housing))) & (self-assessed health/mental health/ depression)”	93	9
2	*“(heat stress/air conditioning) & (wellbeing/ depression/ mental health)”*	NA **	*1*
***TRANSPORT***			
1	“(air pollution/noise) & (mental health/depression)”	54	19
2 ***	“(public transport/exercise/physical activity) & (mental health/anxiety/depression)”	568	1 (public transport related)
3	“(commut */transport mode/public transport/active transport) & (social capital/community/social network /volunteer */cultur *)”	51	15
*4*	*“(accessibility/exclusion) & transport & wellbeing”*	*NA*	*7*
*5*	*“(green/environment/sustainable) & wellbeing”*	*NA*	*7*
*6*	*“(affordability/ frugality) & (wellbeing/depression/mental health)”*	*NA*	*5*
***INDUSTRY***			
1	“(unemployment/employment/job) & (greenhouse gas)”	49	0

Notes: * In some searches these search terms were modified in order to acquire more papers if papers discovered implied other search terms would be beneficial; ** NA (and italic font) indicate “not applicable”—these were supplemental searches where papers were only added to the database if they were found to contain relevant quantitative assessment of links; *** Transport search 2 papers were only considered further if they related to public transport as the relationship between physical activity and mental health was considered established.

Searches for articles on damp and thermal comfort and wellbeing generated reasonable numbers of papers on cold and damp housing. Due to a lack of papers found on uncomfortably hot housing a supplemental search was conducted but only one paper with quantification of links between too hot housing and wellbeing was found.

Transport policy searches included firstly a search on noise and air pollution and wellbeing. Several studies had attempted quantification of links. Secondly there were searches regarding transport mode and wellbeing, only one reference considered public transport and physical activity, via walking to public transport and only one qualitative reference on public transport and mental health was found. More references were found through searches for active transport and social capital. References on the association between accessibility and mental health were found through supplementary searches but mostly through searches of reference lists.

Transport related GHG reduction policies include encouraging use of alternative transport fuels and in the conceptual model this was posed to affect subjective wellbeing through air pollution alone. Additional supplementary searches were made looking at other consequences of these policies that might affect subjective wellbeing. It was thought that biofuels might lead to subjective wellbeing benefits through adoption of a greener lifestyle and that electric cars might make motoring less affordable.

Although there were 49 references in the energy and employment database, no references presented generalizable quantitative assessments of the relationship between GHG reduction policies and changes in employment industry or power generation.

A selection of findings, with some notes on their potential for quantification of wellbeing effects, is provided in Table 2. We assessed study quality through study design (cross-sectional or longitudinal), sample size, sample location (city, region, or country wide for example) and the statistic that was presented. The choice of statistic affects whether results provided could be used by policy makers to predict the results of implementing a policy on wellbeing in their jurisdiction; ideally we were looking for exposure response functions (ERFs), with the next preference being rate ratios; then odds ratios and the least useful being percentages or proportions. 

In traditional HIA, ERFs are used to show that, for example, a reduction in damp in x% homes will lead to a decrease of y% in asthma cases. However, only few ERFs have been estimated as yet between city conditions and wellbeing outcomes (such as between noise and noise annoyance), which makes it very difficult to carry out wellbeing assessments similar to the methods applied for HIA. In addition, there are methodological concerns about those that have been estimated, particularly about the direction of causality [101,139].

In general, the literature search revealed profound weaknesses in existing quantitative approaches to wellbeing measurement. There were conceptual problems with direction of causation, for example does walking increase social capital or are individuals with higher levels of social capital more likely to walk [101,140,141,142,143,144,145,146,147,148,149]? Does damp increase depression or are depressed people less likely to deal with damp or more likely to report housing problems? The majority of the studies identified were cross-sectional and thus unable to explore these connections in sufficient depth [150]. It is likely, however, that bi-directional causal models would be needed, involving feedback mechanisms between causes and effects; this would be more challenging than a traditional one-direction causal model [118].

**Table 2:** Selected examples of wellbeing implications of urban GHG policy implications and potential for quantification of associations *.

*Policies*	*Implications*	*Objective Wellbeing Aspects Explored*	*Subjective Wellbeing Aspects Explored*	*Notes on Potential for Quantification*
**BUILDINGS**				
Tightening building envelope & improving insulation	Reduced air flow and reduced heat loss through building envelope	**Mould and damp**	**Depression** **Mental health** **Self-assessed health** **Satisfaction with indoor air quality**	Some evidence of a relationship found [98,150,151,152,153,154,155,156,157,158] but many studies are cross sectional or based in the UK (particularly the West of Scotland where there is a particular concentration of damp housing and disadvantage). Some odds ratios available.
		**Thermal comfort**	**Depression** **Mental health** **Residential satisfaction** **Self-assessed health**	Most literature appears to have focused on insufficiently warm housing [152,153,154,155,156,157,158] whereas the combination of global warming and increased ventilation may lead to insufficiently cool housing [159]. Some odds ratios available. Differentiation of the effects of cold and damp is difficult.
**TRANSPORT**				
Tolls & Parking restrictions	Reduce private car use	**Air pollution**	**Depression** **Suicide** **Mental Health**	Fairly consistent findings [160,161,162] and one Canadian research team has provided relative risks [132,163,164,165]. However there are many differences by time of year, type of air pollution and gender. Some relative risks available.
		**Air pollution**	**Annoyance**	ERFs developed for Europe [166,167] but direction of causality could be an issue [139].
		**Noise**	**Annoyance** **Sleep disturbance** **Mental health** **Depression** **Satisfaction**	Fairly consistent associations [98,118,121,122,168,169,170,171,172,173]. ERFs developed for annoyance and sleep disturbance [174,175]. Again direction of causality could be an issue [139].
		**Accessibility**	**Mental health**	There is a little, mostly descriptive, research on accessibility and wellbeing mostly from one Australian research team [99,176,177,178,179,180,181] which is suggestive of an association.
Biofuels	**Leading a green lifestyle**		**Life satisfaction** **Happiness** **Social wellbeing**	A consistent relationship found between leading a green lifestyle and wellbeing but studies have tended to use scales rather than dichotomous outcomes so the search did not find any ratios—generalising from the particular scales used is difficult [182,183,184,185].
Electric cars	Cars are less affordable	**Affordability**	**Stress** **Depression** **Happiness**	Studies on affordability and wellbeing are inconclusive [99,182,183,186,187,188]. Again outcomes tend to be on a continuous scale so ratios were not found.
Promotion of public transport		**Use of public transport**	**Mental health**	A few qualitative & descriptive studies [189] or benefits via extra walking [190]
Cycle paths and foot paths	**More walking and cycling**	**Social capital:** **informal social networks,** **community participation,** **trust, voting**		Studies tend to be cross sectional so difficult to tell the direction of causation [101,140,141,142,143,144,145,146,147,148,149]. Odds ratios are available.
		**Physical activity levels**	**Mental health** **Wellbeing**	There is a vast literature in this area (e.g., [101,191,192,193,194,195,196,197,198,199]).
**INDUSTRY**				
Industries encouraged/ discouraged by city	**Change in employment due to cc policies e.g., Power generation**	**Unemployment**	Mental health	One European study has looked at climate change policies and unemployment but the results were not presented in a generalizable manner [113] and other papers are descriptive [200,201,202,203].

Note: * Shaded cells with bold font depict relationships which were assessed for quantification.

Secondly there were many concerns about generalizability. Some studies only provided proportions rather than odds ratios or relative risks [138,140,141]; research on some topics has often concentrated on a particular geographical area with a particular culture or weather conditions; additionally many different wellbeing outcomes were measured. Most studies used measures of life satisfaction or mental health, particularly depression rather than subjective wellbeing, often through a plethora of scales rather than a dichotomous measure, so it is not possible to tell the extent to which a score or a change on a particular scale could be generalized to another [167,168,172]. 

Thirdly it is difficult to distinguish or disentangle concurrent effects, and for example, to establish the extent to which higher levels of depression are related to damp or cold housing, or to generally low socioeconomic status that may be associated with low-quality housing with a higher likelihood for dampness or inadequate thermal performance [93].

Fourthly many studies had not focused on the most relevant aspects, for example most of the literature concentrated on the impact of insufficiently warm housing rather than over warm housing which may be a more pressing issue in settings with reduced ventilation and increasing temperatures. Furthermore some topics, such as the effect of changing power generation source on unemployment, have received little attention within existing research. 

In conclusion, there were many relevant studies on wellbeing or wellbeing-related outcomes of urban environmental conditions, but they did not provide the quality of evidence needed for underpinning a wellbeing assessment of specific urban interventions. Few ERF values were found and even when they were identified there were concerns over their validity. Thus new approaches are needed to assess wellbeing effects of policy interventions. Such a new methodology should involve firstly quantification of subjective wellbeing in relation to specific urban conditions to derive risk ratios and allow for wellbeing assessments to be done in the same way as HIAs. If such risk ratios cannot be identified or modelled, other and potentially more crude or basic measures might have to be considered to enable a first, indicative assessment of potential wellbeing impact of urban policies. For such approaches, all data sources providing information on urban conditions and wellbeing could be of interest. Secondly, any new research program should try to take into account that ERFs may be more varying and context related for wellbeing than health outcomes and in the methodology allow for vulnerability across specific groups whose priorities and needs may be completely different and understand and encompass priorities of different stakeholders (both from wellbeing and policy perspectives).

We recommend urban policy-makers take the following steps, based on those underpinning traditional HIA exercises. Firstly baseline levels of subjective wellbeing and city conditions should be determined, perhaps through use or modification of already-existing data and survey methods such as the EQLS [45] which was conducted in 2003, 2007 and 2012 and includes measures of housing conditions, perceived air quality, traffic and greenspace together with measures of subjective wellbeing (WHO-5 (Note that some of the translations of the WHO-5 used in EQLS are different from the translations specified by the developers of WHO-5), happiness and life satisfaction). Alternatively cities with sufficient resources may wish to conduct their own wellbeing survey into which tools targeting subjective wellbeing and life satisfaction should also be embedded (see [33,34,35] for guidance on various wellbeing measures.) 

Secondly estimates are needed regarding the potential effects of policies on urban living conditions (objective wellbeing). The URGENCHE project is developing strategies for estimating such effects [1,204,205]. This includes, for instance, the relationship between change in traffic flow (via, for example, the implementation of a congestion charge) and air pollution. 

Thirdly estimates are needed regarding the relationship between city conditions and subjective wellbeing. For some relationships estimates could perhaps be developed (Table 2) although, given the issues identified above, they should be used with caution. For other wellbeing effects, alternative ways must be found to quantify the effect of a given policy on urban conditions and associated changes in subjective wellbeing at population level; for an example see work by Rehdanz and Maddison [131]. 

## 5. Conclusions

In this paper we discuss the theoretical aspects that are to be considered when linking wellbeing to urban policies. In brief, we suggest that urban policies should be evaluated within a broad health perspective that includes wellbeing. Wellbeing assessment requires a consistent conceptual model that can then also enable prioritization of interventions. While we have chosen the WHO-5 scale to describe our conceptual model on environmental influences on wellbeing, other wellbeing approaches may be as reasonable and indeed we need to know the effects of policies on satisfaction with various life domains and overall (as recommended by the WHO Regional Office for Europe Health 2020 policy monitoring framework [49,50]), in addition to developing further understanding of the theoretical and empirical links behind policies’ consequences for psychological functioning and hedonic and eudemonic wellbeing.

Here, we have proposed a conceptual model of wellbeing that should make understanding the concept of wellbeing and effects on wellbeing from policies to reduce greenhouse gas emissions easier for local policy makers. Care must be taken when measuring wellbeing to differentiate subjective wellbeing (positive affect) from objective wellbeing (personal, social and environmental conditions that are likely to engender feelings of subjective wellbeing). Dangers of not separating objective and subjective wellbeing may include assumptions by policy makers (and citizens) that high levels of objectively measurable assets are desirable when the literature on subjective wellbeing and socioeconomic status suggests that although disadvantage does reduce subjective wellbeing, affluence does not increase subjective wellbeing [42]. Moreover sustainability issues imply that overconsumption will lead to objective and subjective wellbeing declines for all long term [206].

Climate change policies include buildings, transport, and energy generation interventions and they all theoretically have implications for wellbeing. However there remains a lack of thorough research exploring such interconnections. This lack of attention means that as yet it appears not possible to conduct wellbeing assessments equivalent in rigor to a traditional HIA. However, the compilation of literature reported here did not conform to the stipulations of a systematic review and we recommend that systematic reviews of each of the conceptualized associations are conducted to contribute to future wellbeing assessments of policies. Within these, searches of other databases, such as Cochrane and Psychinfo, should be considered.

Furthermore it is important to acknowledge the local context given the variations found in the wellbeing scores in different settings and cultures, and that wellbeing and the effects of policies are likely to differ by socioeconomic status. The co-existence of environmental exposures and socio-economic factors, known for some agents and some health effects, involve synergistic interactions; this phenomenon is poorly understood even for physical agents and “hard” health outcomes, so its occurrence in the domain of wellbeing is highly speculative. However, because wellbeing involves perceived health, acceptability of risks and ability to cope with such risks, it can be expected that socio-economic factors such as education may play an important role.

Risk estimates, as well as prevalence differences, can be used to provide some sense of the potential impacts. Depending on the intervention and mechanism, variations of the assessment chain and quantification are possible. These can be used to develop a framework for assessing health and wellbeing effects of policies in order develop priorities for urban policy. 

## References

[1] Asikainen A., Savastola M., Parjala E., Kettunen T., Nittynen M., Tuomisto J. URGENCHE WP10: Health Effect Assessment of CO2 Emission Reduction Methods in City of Kuopio. http://www.kuopio.fi/c/document_library/get_file?uuid=990128c5-6c34-4320-b20e-cc1ff8f7d98f&groupId=12141.

[2] Holder M.D. (2012). Happiness in Children: Measurement, Correlates and Enhancement of Positive Subjective Well-Being.

[3] ONS (2012). Measuring National Wellbeing, the Natural Environment. http://www.ons.gov.uk/ons/rel/wellbeing/measuring-national-well-being/natural-environment/art-the-natural-environment.html#tab-Introduction.

[4] Thomas F., Sabel C.E., Morton K., Hiscock R., Depledge M.H. (2014). Extended impacts of climate change on health and wellbeing. Environ. Sci. Policy.

[5] Pridmore A., Miola A. Public Acceptability of Sustainable Transport Measures: A Review of the Literature. Discussion Paper.

[6] WHO Regional Office for Europe Measurement of and Target Setting for Well-Being: An Initiative by the WHO Regional Office for Europe. http://www.euro.who.int/__data/assets/pdf_file/0009/181449/e96732.pdf?ua=1.

[7] Oxfam (2012). Oxfam Human Kind Index: A New Measure of Scotland’s Prosperity. http://policy-practice.oxfam.org.uk/~/media/Files/policy_and_practice/poverty_in_uk/HKI/HKI%20results%20April%202012.ashx.

[8] NEF Happy Planet Index. http://www.happyplanetindex.org/.

[9] WHO Mental Health: A State of Well-Being. http://www.who.int/features/factfiles/mental_health/en/index.html.

[10] GOV.UK, Cabinet Office and Prime Minister’s Office PM Speech on WellbeingLondon.

[11] ONS (2011). Measuring National Wellbeing: A Discussion Paper on Domains and Measures. http://www.ons.gov.uk/ons/rel/wellbeing/measuring-national-well-being/discussion-paper-on-domains-and-measures/measuring-national-well-being---discussion-paper-on-domains-and-measures.html#tab-Introduction.

[12] Williamson T., Casullo A., Thurow J. (2013). How deep is the distinction between a priori and a posteriori knowledge. The a Priori in Philosophy.

[13] Huppert F.A., Cooper C.L. (2014). Wellbeing: A Complete Reference Guide, Interventions and Policies to Enhance Wellbeing.

[14] Taylor D. (2011). Wellbeing and welfare: A psychosocial analysis of being well and doing well enough. J. Soc. Policy.

[15] Dodge R., Daly A.P., Huyton J., Sanders L.D. (2012). The challenge of defining wellbeing. Int. J. Wellbeing.

[16] Carlisle S., Hanlon P. (2007). The complex territory of well-being: contestable evidence, contentious theories and speculative conclusions. J. Public Mental Health.

[17] Carlisle S., Hanlon P. (2008). “Well-being” as a focus for public health? A critique and defence. Crit. Public Health.

[18] Carlisle S., Henderson G., Hanlon P.W. (2009). “Wellbeing”: A collateral casualty of modernity?. Soc. Sci. Med..

[19] WHO Regional Office for Europe Measurement of and Target Setting for Well-Being: An Initiative by the WHO Regional Office for Europe. http://www.euro.who.int/__data/assets/pdf_file/0009/181449/e96732.pdf?ua=1.

[20] Richard E., Diener E. (2009). Personality and Subjective Wellbeing. http://link.springer.com/chapter/10.1007%2F978-90-481-2350-6_4.

[21] Dolan P., Metcalfe R. (2012). Measuring subjective wellbeing: Recommendations on measure for use by national governments. J. Soc. Policy.

[22] Huppert F.A., So T.T. (2013). Flourishing across europe: Application of a new conceptual framework for defining well-being. Soc. Indic Res..

[23] Ryan R.M., Deci E.L. (2001). On happiness and human potentials: A review of research on hedonic and eudaimonic well-being. Annu. Rev. Psychol..

[24] (1948). Constitution of the World Health Organisation.

[25] Saylor C. (2004). The circle of health: A health definition model. J. Holist. Nurs..

[26] Tennant R., Hiller L., Fishwick R., Platt S., Joseph S., Weich S., Parkinson J., Secker J., Stewart-Brown S. (2007). The warwick-edinburgh mental well-being scale (WEMWBS): Development and UK validation. Health Qual. Life Outcomes.

[27] Dreger S., Buck C., Bolte G. (2014). Material, psychosocial and sociodemographic determinants are associated with positive mental health in Europe: A cross-sectional study. BMJ Open.

[28] Bech P., Olsen L.R., Kjoller M., Rasmussen N.K. (2003). Measuring well-being rather than the absence of distress symptoms: A comparison of the SF-36 mental health subscale and the who-five well-being scale. Int. J. Methods Psychiatr. Res..

[29] Allin P., Hand D.J. (2014). The Wellbeing of Nations: Meaning, Motive and Measurement.

[30] (2013). Comprehensive Mental Health Action Plan 2013–2020.

[31] French D., Browning C., Kendig H., Luszcz M., Saito Y., Sargent-Cox K., Anstey K. (2012). A simple measure with complex determinants: Investigation of the correlates of self-rated health in older men and women from three continents. BMC Public Health.

[32] Atkinson S., Joyce K.E. (2011). The place and practices of well-being in local governance. Environ. Plann. C: Gov. Policy.

[33] OECD OECD Guidelines on Measuring Subjective Well-being. http://dx.doi.org/10.1787/9789264191655-en.

[34] Rondinella T., Signore M., Fazio D., Calza M.G., Righi A., E-Frame (Istat) (2014). Map on Policy Use of Progress Indicators [Draft]. Deliverable 11.1.

[35] European Comission (Eurostat) Quality of Life (QoL)—Context. http://epp.eurostat.ec.europa.eu/portal/page/portal/gdp_and_beyond/quality_of_life/context.

[36] Psychiactric Research Unit at the Medical Health Centre North Zealand WHO-Five Well-being Index (WHO-5). http://www.who-5.org/.

[37] Heun R., Bonsignore M., Barkow K., Jessen F. (2001). Validity of the five-item WHO Well-Being Index (WHO-5) in an elderly population. Eur. Arch. Psychiat. Clin. Neuros..

[38] Saipanish R., Lotrakul M., Sumrithe S. (2009). Reliability and validity of the thai version of the who-five well-being index in primary care patients. Psychiatr. Clin. Neurosci..

[39] Awata S., Bech P., Yoshida S., Hirai M., Suzuki S., Yamashita M., Ohara A., Hinokio Y., Matsuoka H., Oka Y. (2007). Reliability and validity of the japanese version of the world health organization-five well-being index in the context of detecting depression in diabetic patients. Psychiat. Clin. Neurosciences.

[40] Hajos T.R., Pouwer F., Skovlund S.E., Den Oudsten B.L., Geelhoed-Duijvestijn P.H., Tack C.J., Snoek F.J. (2013). Psychometric and screening properties of the WHO-5 well-being index in adult outpatients with type 1 or type 2 diabetes mellitus. Diabetic Med..

[41] Shea S., Skovlund S.E., Bech P., Kalo I., Home P. (2003). Routine assessment of psychological well-being in people with diabetes-validation of the WHO-5 well-being index in six countries. Diabetologia.

[42] Tov W., Au E. Comparing Well-Being Across Nations: Conceptual and Empirical Issues. http://ink.library.smu.edu.sg/cgi/viewcontent.cgi?article=2405&context=soss_research&sei-redir=1&referer=http%3A%2F%2Fscholar.google.co.uk%2Fscholar_url%3Fhl%3Den%26q%3Dhttp%3A%2F%2Fink.library.smu.edu.sg%2Fcgi%2Fviewcontent.cgi%253Farticle%253D2405%2526context%253Dsoss_research%26sa%3DX%26scisig%3DAAGBfm3Muoasw4Wkcz0HYwo_Al_BN5xHMw%26oi%3Dscholarr%26ei%3DIeF0VNrZOOWP7AbonYDIBA%26ved%3D0CCEQgAMoADAA#search=%22http%3A%2F%2Fink.library.smu.edu.sg%2Fcgi%2Fviewcontent.cgi%3Farticle%3D2405%26context%3Dsoss_research%22.

[43] Aminzadeh K., Denny S., Utter J., Milfont T.L., Ameratunga S., Teevale T., Clark T. (2013). Neighbourhood social capital and adolescent self-reported wellbeing in New Zealand: A multilevel analysis. Soc. Sci. Med..

[44] Primack B. (2003). The WHO-5 wellbeing index performed the best in screening for depression in primary care. Evid. Based Med..

[45] Eurofound European quality of life surveys (EQLS). http://www.eurofound.europa.eu/surveys/eqls/index.htm.

[46] NHS Health Scotland, University of Warwick, University of Edinburgh The Warwick-Edinburgh Mental Well-being Scale (WEMWBS). http://www.experiential-researchers.org/instruments/leijssen/WEMWBS.pdf.

[47] Stewart-Brown S., Tennant A., Tennant R., Platt S., Parkinson J., Weich S. (2009). Internal construct validity of the warwick-edinburgh mental well-being scale (WEMWBS): A rasch analysis using data from the scottish health education population survey. Health Qual. Life Outcomes.

[48] Keyes C. Mental Well-Being: International Contributions to the Study of Positive Mental Health. http://books.google.co.uk/books?id=_Yv5_LMmPL8C&pg=PA148&lpg=PA148&dq=SWEMWBS&source=bl&ots=7Bc2mXMC2b&sig=LzNh_eZkKT-y4XewSW-IoESFfJQ&hl=en&sa=X&ei=1T9nUc3aAeOx0QW-y4GwAg&ved=0CGAQ6AEwCA#v=onepage&q&f=false.

[49] WHO Regional Office for Europe Joint Meeting of Experts on Targets and Indicators for Health and Well-Being in Health 2020. http://www.euro.who.int/__data/assets/pdf_file/0003/186024/e96819.pdf?ua=1.

[50] WHO Regional office for Europe Health 2020 Targets, Indicators and Monitoring Framework. http://www.euro.who.int/__data/assets/pdf_file/0008/195389/63wd08e_Health-2020-targets-3.pdf?ua=1.

[51] Diener E., Emmons R.A., Larsen R.J., Griffin S. (1985). The satisfaction with life scale. J. Pers. Assess..

[52] Pavot W., Diener E., Colvin C.R., Sandvik E. (1991). Further validation of the satisfaction with life scale: Evidence for the cross-method convergence of well-being measures. J. Pers. Assess..

[53] Diener E. What is Subjective Well-Being (SWB)?. http://internal.psychology.illinois.edu/~ediener/faq.html#SWB.

[54] Pavot W., Diener E. (2013). Happiness Experienced: The Science of Subjective Well-Being. http://www.oxfordhandbooks.com/10.1093/oxfordhb/9780199557257.001.0001/oxfordhb-9780199557257-e-010.

[55] Coughlan S. (2012). Ofsted Plans to Scrap “satisfactory” Label for Schools. BBC News.

[56] Nieboer A., Lindenberg S., Boomsma A., Bruggen A.V. (2005). Dimensions of well-being and their measurement: The SPF-IL scale. Soc. Indic Res..

[57] ONS Subjective Well-Being Survey User Guide: 12 Month Dataset. UK Data Archive Study Group Number 33376—Annual Population Survey: Special Licence Access. https://www.google.co.uk/url?sa=t&rct=j&q=&esrc=s&source=web&cd=1&cad=rja&uact=8&ved=0CCMQFjAA&url=http%3A%2F%2Fwww.ons.gov.uk%2Fons%2Fguide-method%2Fmethod-quality%2Fspecific%2Fsocial-and-welfare-methodology%2Fsubjective-wellbeing-survey-user-guide%2Fsubjective-well-being-survey-user-guide--12-month-dataset---download-version.pdf&ei=b-J0VP-mB4LW7AaZ2IH4BA&usg=AFQjCNE5xD3IVf1yg7kEACzAnzsmBdLDXA&bvm=bv.80185997,d.ZGU.

[58] Dillman D.A., Smyth J.D., Christian L.M. (2009). Internet, Mail, and Mixed-Mode Surveys : The Tailored Design Method.

[59] Miller G.A. (1956). The magical number seven plus or minus two: Some limits on our capacity for processing information. Psychol. Rev..

[60] Cummins R.A. (2010). Subjective wellbeing, homeostatically protected mood and depression: A synthesis. J. Happiness Stud..

[61] ISOCARP (2010). Livable Cities in a Rapidly Urbanizing World.

[62] WHO Regional Office for Europe Second Joint Meeting of Experts on Targets and Indicators for Health and Well-Being in Health 2020. http://www.euro.who.int/__data/assets/pdf_file/0008/253673/Meeting-Report-April-meeting-final-WEB.pdf?ua=1.

[63] OECD Better Life Index. http://www.oecdbetterlifeindex.org/.

[64] Gallup Gallup Healthways Wellbeing Index. http://www.well-beingindex.com/.

[65] ISTAT, CNEL BES 2013 Report: Equitable and Sustainable Wellbeing in Italy. http://www.misuredelbenessere.it/index.php?id=48.

[66] Horsman J., Furlong W., Feeny D., Torrance G. (2003). The health utilities index (hui^®^): Concepts, measurement properties and applications. Health Qual. Life Outcomes.

[67] Amiel M., Godefroy P., Lollivier S. (2013). Quality of Life and Well-Being Often Go Hand in Hand. http://www.insee.fr/en/themes/document.asp?ref_id=ip1428.

[68] ONS National Wellbeing. http://www.ons.gov.uk/ons/guide-method/user-guidance/well-being/index.html.

[69] Boelhouwer J. Wellbeing in the Netherlands: the SCP Life Situation Index Since 1974. www.scp.nl/english/dsresource?objectid=25936&type=org.

[70] Ministry of the Interior Netherlands Leefbaarometer. http://www.leefbaarometer.nl/.

[71] Scott K., Bell D. (2013). Trying to measure local well-being: Indicator development as a site of discursive struggles. Environ. Plann. C: Govern. Policy.

[72] Thompson S., Marks N., Jackson T. Well-Being and Sustainable Development. http://www.oxfordhandbooks.com/view/10.1093/oxfordhb/9780199557257.001.0001/oxfordhb-9780199557257-e-038.

[73] Burchell B. (2011). A temporal comparison of the effects of unemployment and job insecurity on wellbeing. Sociol. Res. Online.

[74] Dolan P., Peasgood T., Dixon A., Knight M., Phillips D., Tsuchiya A., White M. (2006). Research on the Relationship between Well-Being and Sustainable Development.

[75] Schneider R.J. (2013). Theory of routine mode choice decisions: An operational framework to increase sustainable transportation. Transp. Policy.

[76] O’Campo P., Salmon C., Burke J. (2009). Neighbourhoods and mental well-being: What are the pathways?. Health Place.

[77] Brereton F., Clinch J., Ferreira S. (2008). Happiness, gegraphy and the environment. Ecol. Econ..

[78] Wong C.K., Wong K.Y., Mok B.H. (2006). Subjective well-being, societal condition and social policy—The case study of a rich chinese society. Soc. Indic Res..

[79] Diener E., Suh E.M. (2000). Culture and Subjective Well-Being.

[80] Atkinson S., Fuller S., Painter J. (2012). Wellbeing and Place.

[81] Costanza R., Fisher B., Ali S., Beer C., Bond L., Boumans R., Danigelis N.L., Dickinson J., Elliott C., Farley J. (2007). Quality of life: An approach integrating opportunities, human needs, and subjective well-being. Ecol. Econ..

[82] Van Kamp I., Leidelmeijer K., Marsman G., de Hollander A. (2003). Urban environmental quality and human well-being: Towards a conceptual framework and demarcation of concepts; A literature study. Landsc. Urban Plann..

[83] Aslam A., Corrado L. (2012). The geography of well-being. J. Econ. Geogr..

[84] Pacione M. (2003). Urban environmental quality and human wellbeing: A social geographical perspective. Landsc. Urban Plann..

[85] Weissbecker I. (2011). Climate change and human well-being. Global Challenges and Opportunities.

[86] Hanratty B., Farmer S. (2012). Editorial: The new uk focus on well-being: What will it mean for tackling social inequalities in health?. J. Public Health.

[87] Fleuret S., Atkinson S. (2007). Wellbeing, health and geography: A crictical review and research agenda. N. Zeal. Geogr..

[88] Reardon L., Abdallah S. (2013). Well-being and transport: Taking stock and looking forward. Transp. Rev..

[89] Putnam R. (1995). Bowling alone: America’s declining social capital. J. Democr..

[90] Zhou Y.-C. (2006). The action logic between social capital and harmonious society construction. Acad. Explor..

[91] WHO WHOQOL-BREF: Introduction, Administration, Scoring and Generic Version of the Assessment—Field Trial Version. http://www.who.int/mental_health/media/en/76.pdf.

[92] Schmidt S., Mühlan H., Power M. (2006). The EUROHIS-QOL 8-item index: Psychometric results of a cross-cultural field study. Eur. J. Public Health.

[93] Van Beuningen J., de Jonge T. The Personal Wellbeing Index: Construct Validity for the Netherlands. http://www.cbs.nl/NR/rdonlyres/F0C01716-3E08-4A6E-AC30-9234A5EFC5FD/0/2011x1024art.pdf.

[94] International Wellbeing Group (2013). Personal Wellbeing Index-Adult (PWI-A). http://www.deakin.edu.au/research/acqol/instruments/wellbeing-index/pwi-a-english.pdf.

[95] Parfitt D. (1984). Reasons and Persons.

[96] Thomson H., Petticrew M., Douglas M. (2003). Health impact assessment of housing improvements: Incorporating research evidence. J. Epidemiol. Commun. Health.

[97] (2011). Health in the Green Economy: Health Co-Benefits of Climate Change Mitigation in the Housing Sector.

[98] Guite H., Clark C., Ackrill G. (2006). The impact of the physical and urban environment on mental well-being. Public Health.

[99] Delbosc A. (2012). The role of well-being in transport policy. Transp. Policy.

[100] International Institute for Democracy and Electoral Assistance Voter Turnout in Western Europe Since 1945. http://www.idea.int/publications/voter_turnout_weurope/upload/Full_Reprot.pdf.

[101] Addy C.L., Wilson D.K., Kirtland K.A., Ainsworth B.E., Sharpe P., Kimsey D. (2004). Associations of perceived social and physical environmental supports with physical activity and walking behavior. Amer. J. Public Health.

[102] Keniger L.E., Gaston K.J., Irvine K.N., Fuller R.A. (2013). What are the benefits of interacting with nature?. Int. J. Environ. Res. Public Health.

[103] Dur F., Yigitcanlar T., Bunker J. (2014). A spatial-indexing model for measuring neighbourhood-level land-use and transport integration. Environ Plan B-Plan Design.

[104] Gabriel Z., Bowling A. (2004). Quality of life from the perspectives of older people. Ageing Soc..

[105] Pearce J., Witten K., Hiscock R., Blakely T. (2007). Are socially disadvantaged neighbourhoods deprived of health-related community resources?. Int. J. Epidemiol..

[106] Rogers S., Halstead J., Gardner K., Carlson C. (2011). Examining walkability and social capital as indicators of quality of life at the municipal and neighborhood scales. Appl. Res. Qual. Life.

[107] Leyden K.M. (2003). Social capital and the built environment: The importance of walkable neighborhoods. Amer. J. Public Health.

[108] Ball K., Cleland V.J., Timperio A.F., Salmon J., Giles-Corti B., Crawford D.A. (2010). Love thy neighbour? Associations of social capital and crime with physical activity amongst women. Soc. Sci. Med..

[109] Rotko T., Oglesby L., Künzli N., Carrer P., Nieuwenhuijsen M.J., Jantunen M. (2002). Determinants of perceived air pollution annoyance and association between annoyance scores and air pollution (PM_2.5_, NO_2_) concentrations in the european expolis study. Atmos. Environ..

[110] Colvile R.N., Hutchinson E.J., Mindell J.S., Warren R.F. (2001). The transport sector as a source of air pollution. Atmos. Environ..

[111] WHO Regional Office for Europe WHO LARES Final Report Noise Effects and Morbidity. http://www.euro.who.int/__data/assets/pdf_file/0015/105144/WHO_Lares.pdf.

[112] Verheijen E., Jabben J. Effect of Electric Cars on Traffic Noise and Safety. http://www.rivm.nl/bibliotheek/rapporten/680300009.pdf.

[113] European Trade Union Confederation (ETUC) Climate Change and Employment: Impact on Employment of Climate Change and CO_2_ Emission Reduction Measures in the EU-25 to 2030 Brussels, 2007. http://www.unizar.es/gobierno/consejo_social/documents/070201ClimateChang-Employment.pdf.

[114] UNEP (2008). Green Jobs: Towards Decent Work in a Sustainable, Low-Carbon World.

[115] Levy C. A 2020 Low Carbon Economy: A Knowldege Economy Programme Report. http://www.theworkfoundation.com/DownloadPublication/Report/243_lowcarbonFINAL_CORRECTED.pdf.

[116] Despres C. (1991). The meaning of home: Literature review and directions for future research and theoretical development. J. Archit. Plann. Res..

[117] Petticrew M., Kearns A., Mason P., Hoy C. (2009). The sharp study: A quantitative and qualitative evaluation of the short-term outcomes of housing and neighbourhood renewal. BMC Public Health.

[118] Urban J., Máca V. (2013). Linking traffic noise, noise annoyance and life satisfaction: A case study. Int. J. Environ. Res. Public Health.

[119] Birk M., Ivina O., von Klot S., Babisch W., Heinrich J. (2011). Road traffic noise: Self-reported noise annoyance *versus* gis modelled road traffic noise exposure. J. Environ. Monit..

[120] Fyhri A., Klaeboe R. (2009). Road traffic noise, sensitivity, annoyance and self-reported health—A structural equation model exercise. Environ. Int..

[121] Kawada T., Yosiaki S., Yasuo K., Suzuki S. (2003). Population study on the prevalence of insomnia and insomnia-related factors among japanese women. Sleep Med..

[122] Li H.-J., Yu W.-B., Lu J.-Q., Zeng L., Li N., Zhao Y.-M. (2008). Investigation of road-traffic noise and annoyance in Beijing: A cross-sectional study of 4th ring road. Arch. Environ. Occup. Health.

[123] Miedema H.M., Oudshoorn C.G. (2001). Annoyance from transportation noise: Relationships with exposure metrics DNL and DENL and their confidence intervals. Environ. Health Persp..

[124] Ising H., Kruppa B. (2004). Health effects caused by noise: Evidence in the literature form the past 25 years. Noise Health.

[125] Sallis J.F., Glanz K. (2006). The role of built environments in physical activity, eating, and obesity in childhood. Future Child.

[126] Greenfield E.A., Reyes L. (2014). Continuity and change in relationships with neighbors: Implications for psychological well-being in middle and later life. J. Gerontol. Ser. B-Psychol. Sci..

[127] Becchetti L., Ricca E.G., Pelloni A. (2012). The relationship between social leisure and life satisfaction: Causality and policy implications. Soc. Indic Res..

[128] Rodríguez-Pose A., von Berlepsch V. (2014). Social capital and individual happiness in Europe. J. Happiness Stud..

[129] Shenassa E., Liebhaber A., Ezeamama A. (2006). Perceived safety of area of residence and exercise: A pan-european study. Amer. J. Epidemiol..

[130] Welsch H. (2002). Preference over prosperity and pollution: Environmental valuation based on happiness surveys. Kyklos.

[131] Rehdanz K., Maddison D. (2008). Local environmental quality and life-satisfaction in germany. Ecol. Econ..

[132] Szyszkowicz M., Rowe B.H., Colman I. (2009). Air pollution and daily emergency department visits for depression. Int. J. Occup. Med. Environ. Health.

[133] Clougherty J.E., Kubzansky L.D. (2010). A framework for examining social stress and susceptibility to air pollution in respiratory health. Ciência Saúde Coletiva.

[134] Downey L., Van Willigen M. (2005). Environmental stressors: The mental health impacts of living near industrial activity. J. Health Soc. Behav..

[135] Pascal M., Pascal L., Bidondo M.-L., Cochet A., Sarter H., Stempfelet M., Wagner V. (2013). A review of the epidemiological methods used to investigate the health impacts of air pollution around major industrial areas. J. Environ. Public Health.

[136] McManus S., Mowlam A., Dorsett D., Stansfeld S., Clark C., Brown V., Wollny I., Rahim N., Morrell G., Graham J. (2012). Mental Health in Context: The National Study of Worksearch and Wellbeing.

[137] Weich S., Brugha T., King M., McManus S., Bebbington P., Jenkins R., Cooper C., McBride O., Stewart-Brown S. (2011). Mental well-being and mental illness: Findings from the adult psychiatric morbidity survey for england 2007. Brit. J. Psychiat..

[138] Gili M., Roca M., Basu S., McKee M., Stuckler D. (2013). The mental health risks of economic crisis in Spain: Evidence from primary care centres, 2006 and 2010. Eur. J. Public Health.

[139] Persson R., Bjork J., Ardo J., Albin M., Jakobsson K. (2007). Trait anxiety and modeled exposure as determinants of self-reported annoyance to sound, air pollution and other environmental factors in the home. Int. Arch. Occup. Environ. Health.

[140] Lindstrom M. (2011). Social capital, desire to increase physical activity and leisure-time physical activity: A population-based study. Public Health.

[141] Renalds A., Smith T.H., Hale P.J. (2010). A systematic review of built environment and health. Fam. Commun. Health.

[142] Nguyen D. (2010). Evidence of the impacts of urban sprawl on social capital. Environ. Plan. B-Plan. Des..

[143] Hopkins D., Williamson T. (2012). Inactive by design? Neighborhood design and political participation. Polit. Behav..

[144] Richard L., Gauvin L., Gosselin C., Laforest S. (2009). Staying connected: Neighbourhood correlates of social participation among older adults living in an urban environment in Montreal, Quebec. Health Promot. Int..

[145] Wood L., Frank L.D., Giles-Corti B. (2010). Sense of community and its relationship with walking and neighborhood design. Soc. Sci. Med..

[146] Wilkerson A., Carlson N.E., Yen I.H., Michael Y.L. (2012). Neighborhood physical features and relationships with neighbors: Does positive physical environment increase neighborliness?. Environ. Behav..

[147] Toit L.D., Cerin E., Leslie E., Owen N. (2007). Does walking in the neighbourhood enhance local sociability?. Urban Studies.

[148] Poortinga W. (2006). Perceptions of the environment, physical activity, and obesity. Soc. Sci. Med..

[149] Hanibuchi T., Kondo K., Nakaya T., Shirai K., Hirai H., Kawachi I. (2012). Does walkable mean sociable? Neighborhood determinants of social capital among older adults in Japan. Health Place.

[150] Shenassa E.D., Daskalakis C., Liebhaber A., Braubach M., Brown M. (2007). Dampness and mold in the home and depression: An examination of mold-related illness and perceived control of one’s home as possible depression pathways. Amer. J. Public Health.

[151] Packer C.N., Stewartbrown S., Fowle S.E. (1994). Damp housing and adult health-results from a life-style study in Worcester, England. J. Epidemiol. Commun. Health.

[152] WHO Regional Office for Europe Large Analysis and Review of European Housing and Health Status (LARES): Preliminary Overview. http://www.euro.who.int/en/health-topics/environment-and-health/Housing-and-health/activities/the-large-analysis-and-review-of-european-housing-and-health-status-lares-project.

[153] Evans J., Hyndman S., Stewart-Brown S., Smith D., Petersen S. (2000). An epidemiological study of the relative importance of damp housing in relation to adult health. J. Epidemiol. Commun. Health.

[154] Hopton J.L., Hunt S.M. (1996). Housing conditions and mental health in a disadvantaged area in scotland. J. Epidemiol. Commun. Health.

[155] Blackman T., Harvey J., Lawrence M., Simon A. (2001). Neighbourhood renewal and health: Evidence from a local case study. Health Place.

[156] Hopton J., Hunt S. (1996). The health effects of improvements to housing: A longitudinal study. Hous. Stud..

[157] Hyndman S.J. (1990). Housing dampness and health amongst british bengalis in east London. Soc. Sci. Med..

[158] Butler S., Williams M., Tukuitonga C., Paterson J. (2003). Problems with damp and cold housing among pacific families in New Zealand. N. Z. Med. J..

[159] Tawatsupa B., Yiengprugsawan V., Kjellstrom T., Seubsman S.-A., Sleigh A., The Thai Cohort Study Team (2012). Heat stress, health and well-being: Findings from a large national cohort of Thai adults. BMJ Open.

[160] Welsch H. (2006). Environment and happiness: Valuation of air pollution using life satisfaction data. Ecol. Econ..

[161] Menz T. (2011). Do people habituate to air pollution? Evidence from international life satisfaction data. Ecol. Econ..

[162] Lim Y.-H., Kim H., Kim J.H., Bae S., Park H.Y., Hong Y.-C. (2012). Air pollution and symptoms of depression in elderly adults. Environ. Health Persp..

[163] Szyszkowicz M., Willey J.B., Grafstein E., Rowe B.H., Colman I. (2010). Air pollution and emergency department visits for suicide attempts in Vancouver, Canada. Environ. Health Insights.

[164] Szyszkowicz M. (2007). Air pollution and emergency department visits for depression in edmonton, Canada. Int. J. Occup. Med. Environ. Health.

[165] Szyszkowicz M., Tremblay N. (2011). Case-crossover design: Air pollution and health outcomes. Int. J. Occup. Med. Environ. Health.

[166] Amundsen A.H., Klaeboe R., Fyhri A. (2008). Annoyance from vehicular air pollution: Exposure-response relationships for Norway. Atmos. Environ..

[167] Klaeboe R., Amundsen A.H., Fyhri A. (2008). Annoyance from vehicular air pollution: A comparison of european exposure-response relationships. Atmos. Environ..

[168] Paunovic K., Jakovljevic B., Belojevic G. (2009). Predictors of noise annoyance in noisy and quiet urban streets. Sci. Total Environ..

[169] Kryter K.D. (2009). Acoustical model and theory for predicting effects of environmental noise on people. J. Acoust. Soc. Amer..

[170] Schram-Bijkerk D., van Kempen E., Knol A.B., Kruize H., Staatsen B., van Kamp I. (2009). Quantitative health impact assessment of transport policies: Two simulations related to speed limit reduction and traffic re-allocation in the Netherlands. Occup. Environ. Med..

[171] Yoshida T., Osada Y., Kawaguchi T., Hoshiyama Y., Yoshida K., Yamamoto K. (1997). Effects of road traffic noise on inhabitants of Tokyo. J. Sound Vibr..

[172] Schreckenberg D., Griefahn B., Meis M. (2010). The associations between noise sensitivity, reported physical and mental health, perceived environmental quality, and noise annoyance. Noise Health.

[173] Dratva J., Zemp E., Dietrich D.F., Bridevaux P.-O., Rochat T., Schindler C., Gerbase M.W. (2010). Impact of road traffic noise annoyance on health-related quality of life: Results from a population-based study. Qual. Life Res..

[174] Miedema H.M.E., Vos H. (1998). Exposure-response relationships for transportation noise. J. Acoust. Soc. Amer..

[175] Miedema H.M., Vos H. (2007). Associations between self-reported sleep disturbance and environmental noise based on reanalyses of pooled data from 24 studies. Behav. Sleep Med..

[176] Stanley J.K., Hensher D.A., Stanley J.R., Vella-Brodrick D. (2011). Mobility, social exclusion and well-being: Exploring the links. Transp. Res. Part A-Policy Pract..

[177] Johnson V., Currie G., Stanley J. (2010). Measures of disadvantage: Is car ownership a good indicator?. Soc. Indicat. Res..

[178] Hurni A., Currie G., Stanley J., Stanley J. (2007). Marginalised groups in western sydney: The experience of sole parents and unemployed young people. No Way to Go: Transport and Social Disadvantage in Australian Communities.

[179] Delbosc A., Currie G. (2011). Exploring the relative influences of transport disadvantage and social exclusion on well-being. Transp. Policy.

[180] Currie G., Delbosc A. (2010). Modelling the social and psychological impacts of transport disadvantage. Transportation.

[181] Olsson L.E., Jakobsson C., Gamble A., Garling T. (2008). The road to happiness? Car use and subjective well-being. Int. J. Psychol..

[182] Corral-Verdugo V., Mireles-Acosta J., Tapia-Fonllem C., Fraijo-Sing B. (2011). Happiness as correlate of sustainable behavior: A study of pro-ecological, frugal, equitable and altruistic actions that promote subjective wellbeing. Hum. Ecol. Rev..

[183] Kasser T., Sheldon K. (2002). What makes for a merry Christmas?. J. Happiness Stud..

[184] Mellan M. (2006). Green and Happy? The Relationship between Personal Well-Being and Environmental Knowledge, Attitudes and Behaviours.

[185] Brown K.W., Kasser T. (2005). Are psychological and ecological well-being compatible? The role of values, mindfulness, and lifestyle. Soc. Indic Res..

[186] Bentley R., Baker E., Mason K. (2012). Cumulative exposure to poor housing affordability and its association with mental health in men and women. J. Epidemiol. Community Health.

[187] Bentley R., Baker E., Mason K., Subramanian S.V., Kavanagh A.M. (2011). Association between housing affordability and mental health: A longitudinal analysis of a nationally representative household survey in australia reply. Amer. J. Epidemiol..

[188] Harkness J., Newman S. (2009). Geographic differences in housing prices and the wellbeing of children and parents. J. Urban Affair..

[189] Jones A., Steinbach R., Roberts H., Goodman A., Green J. (2012). Rethinking passive transport: Bus fare exemptions and young people’s wellbeing. Health Place.

[190] Besser L.M., Dannenberg A.L. (2005). Walking to public transit: Steps to help meet physical activity recommendations. Amer. J. Prev. Med..

[191] Gatersleben B., Uzzell D. (2007). Affective appraisals of the daily commute-comparing perceptions of drivers, cyclists, walkers, and users of public transport. Environ. Behav..

[192] Wen L.M., Rissel C. (2008). Inverse associations between cycling to work, public transport, and overweight and obesity: Findings from a population based study in Australia. Prev. Med..

[193] Abu-Omar K. (2004). Mental health and physical activity in the European Union. Sozial-Und Praventivmedizin.

[194] Culpepper D., Jevas S., Perkins H. (2004). Predicting symptoms of depression based on self-reported levels of physical activity. Res. Quart. Exercise Sport.

[195] Daley A. (2008). Exercise and depression: A review of reviews. J. Clin. Psychol. Med. Set..

[196] Holley J. (2011). Physical activity and mental health: Reflections from research and implications for practice. Ment. Health Today.

[197] Kirby S. (2005). The positive effect of exercise as a therapy for clinical depression. Nurs. Times.

[198] Deslandes A., Moraes H., Ferreira C., Veiga H., Silveira H., Mouta R., Pompeu F.A., Coutinho E.S., Laks J. (2009). Exercise and mental health: Many reasons to move. Neuropsychobiology.

[199] Rissel C.E. (2009). Active travel: A climate change mitigation strategy with co-benefits for health. N. S. W. Public Health Bull..

[200] Paul K.I., Moser K. (2009). Unemployment impairs mental health: Meta-analyses. J. Vocat. Behav..

[201] Neuwahl F., Loeschel A., Mongelli I., Delgado L. (2008). Employment impacts of eu biofuels policy: Combining bottom-up technology information and sectoral market simulations in an input-output framework. Ecol. Econ..

[202] Babiker M.H., Eckaus R.S. (2007). Unemployment effects of climate policy. Environ. Sci. Policy.

[203] Sonneborn C. (2000). Generating jobs-sustainable energy initiatives deliver more jobs and lower greenhouse gas emissions. Altern. J..

[204] Sarigiannis D.A., Karakitsios S.P., Kermenidou M., Nikolaki S., Zikopoulos D., Semelidis S., Papagiannakis A., Tzimou R. (2014). Total exposure to airborne particulate matter in cities: The effect of biomass combustion. Sci. Total Environ..

[205] Keuken M., Jonkers S., Verhagen H., Perez L., Trüeb S., Okkerse W.-J., Liu J., Pan X., Zheng L., Wang H. (2014). Impact on air quality of measures to reduce CO_2_ emissions from road traffic in Basel, Rotterdam, Xi’an and Suzhou. Atmosp. Environ..

[206] (2010). Global Footprint Network. Ecological Footprint Atlas.

